# 2,2′,2′′,2′′′-[Pyridine-2,6-diylbis(methyl­ene­nitrilo)]tetra­ethanol

**DOI:** 10.1107/S1600536811010919

**Published:** 2011-03-31

**Authors:** Shou-Ying Cao, Cheng-He Zhou

**Affiliations:** a School of Chemistry and Chemical Engineering, Southwest University, Chongqing, 400715, People’s Republic of China

## Abstract

In the crystal structure of the title compound C_15_H_27_N_3_O_4_, the mol­ecule is located on a twofold axis and the asymmetric unit contains one half-mol­ecule, with one N and one C atom lying on the rotation axis. The pyridine ring is the hydrogen-bond acceptor, while two hydroxyl O atoms act as hydrogen-bond donors in intra­molecular O—H⋯N and intermolecular O—H⋯N and O—H⋯O hydrogen bonds, thereby forming a closed hydrogen-bonded cage.

## Related literature

For general background to pyridine, see: Klimesova *et al.* (1990 ▶); Rabasseda *et al.* (1999 ▶) . For the synthesis, see: Fang *et al.* (2010 ▶).
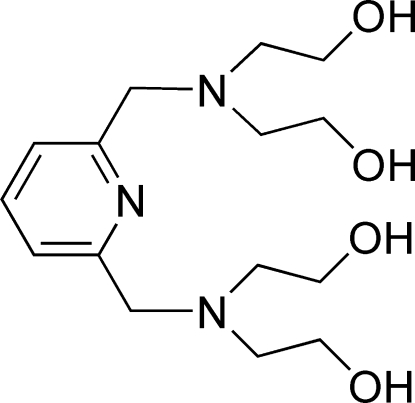


## Experimental

### 

#### Crystal data

C_15_H_27_N_3_O_4_
*M* *_r_* = 313.40
Orthorhombic, 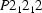

*a* = 9.145 (6) Å
*b* = 10.716 (7) Å
*c* = 8.292 (5) Å
*V* = 812.6 (9) Å^3^
*Z* = 2
Mo *K*α radiation
μ = 0.09 mm^−1^
*T* = 298 K
0.45 × 0.45 × 0.45 mm

#### Data collection

Bruker SMART APEX diffractometer
4239 measured reflections
1511 independent reflections
1441 reflections with *I* > 2σ(*I*)
*R*_int_ = 0.021

#### Refinement

*R*[*F* ^2^ > 2σ(*F* ^2^)] = 0.036
*wR*(*F* ^2^) = 0.098
*S* = 1.08
1511 reflections
104 parameters
1 restraint
H-atom parameters constrained
Δρ_max_ = 0.14 e Å^−3^
Δρ_min_ = −0.17 e Å^−3^

### 

Data collection: *SMART* (Bruker, 2000 ▶); cell refinement: *SAINT* (Bruker, 2000 ▶); data reduction: *SAINT*; program(s) used to solve structure: *SHELXS97* (Sheldrick, 2008 ▶); program(s) used to refine structure: *SHELXL97* (Sheldrick, 2008 ▶); molecular graphics: *SHELXTL* (Sheldrick, 2008 ▶); software used to prepare material for publication: *SHELXTL*.

## Supplementary Material

Crystal structure: contains datablocks I, global. DOI: 10.1107/S1600536811010919/rk2270sup1.cif

Structure factors: contains datablocks I. DOI: 10.1107/S1600536811010919/rk2270Isup2.hkl

Additional supplementary materials:  crystallographic information; 3D view; checkCIF report

## Figures and Tables

**Table 1:** Hydrogen-bond geometry (Å, °)

*D*—H⋯*A*	*D*—H	H⋯*A*	*D*⋯*A*	*D*—H⋯*A*
O2—H2*A*⋯O1^i^	0.82	1.94	2.762 (2)	175
O1—H1*A*⋯N2^i^	0.82	2.74	3.248 (2)	122
O1—H1*A*⋯N2	0.82	2.54	2.935 (2)	111
O1—H1*A*⋯N1	0.82	2.25	3.004 (2)	153

Symmetry code: (i) 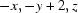.

**Fig. 1.**
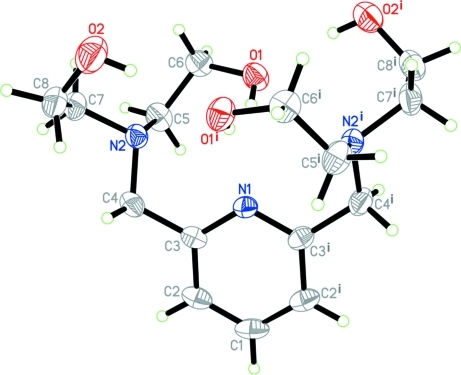
The molecular structure of the title compound, showing the atom–numbering scheme. Displacement ellipsoids are drawn at the 30% probability level. H atoms are presented as a small spheres of arbitrary radius.

## supplementary crystallographic information

### Comment

Pyridine is an important type of nitrogen–containing aromatic heterocycle which is widely used as building block for preparing a variety of drugs, insecticides and herbicides in the pharmaceutical industry (Klimesova *et al.*, 1999; Rabasseda *et al.*, 1999). Recently, our researches have been focused on the development of aromatic ring–derived nitrogen mustards as potential antitumor agents. As part of our work, herein we report the molecular and crystal structures of the title compound as an intermediate for the pyridine–derived nitrogen mustards.

The molecular structure of the title compound is shown in Fig. 1. In the crystal structure of the title compound, C_15_H_27_N_3_O_4_, reveals that the molecule placed on two–fold axis (C1, H1 and N1 atoms placed on twofold axis) and asymmetric unit contains a half molecule. In the molecule, pyridine ring is the hydrogen–bond acceptor, and two hydroxyls which move closer to the pyridine ring *via* O—H···N intramolecular hydrogen bond perform as hydrogen–bond donor, thus the closed hydrogen bond cage has been formed.

### Experimental

The title compound was prepared according to the procedure of Fang *et al.* (2010). Single crystals were grown by slow evaporation of a solution of the title compound in the mixture of dichloromethane and *n*–hexane at room temperature.

### Refinement

All H atoms were placed in idealized positions and treated as riding, with C—H = 0.93Å (CH) or 0.97Å (CH_2_), and *U*_iso_(H) = 1.2*U*_eq_(C); O—H = 0.82Å and *U*_iso_(H) = 1.5*U*_eq_(O).

### Crystal data

**Table:**

C_15_H_27_N_3_O_4_	*F*(000) = 340
*M**_r_* = 313.40	*D*_x_ = 1.281 Mg m^−^^3^
Orthorhombic, *P*2_1_2_1_2	Mo *K*α radiation, λ = 0.71073 Å
Hall symbol: P 2 2ab	Cell parameters from 2637 reflections
*a* = 9.145 (6) Å	θ = 2.5–27.1°
*b* = 10.716 (7) Å	µ = 0.09 mm^−^^1^
*c* = 8.292 (5) Å	*T* = 298 K
*V* = 812.6 (9) Å^3^	Block, colourless
*Z* = 2	0.45 × 0.45 × 0.45 mm

### Data collection

**Table:**

Bruker SMART APEX diffractometer	1441 reflections with *I* > 2σ(*I*)
Radiation source: fine-focus sealed tube	*R*_int_ = 0.021
graphite	θ_max_ = 25.5°, θ_min_ = 2.5°
φ– and ω–scans	*h* = −11→8
4239 measured reflections	*k* = −9→12
1511 independent reflections	*l* = −10→8

### Refinement

**Table:**

Refinement on *F*^2^	Secondary atom site location: difference Fourier map
Least-squares matrix: full	Hydrogen site location: inferred from neighbouring sites
*R*[*F*^2^ > 2σ(*F*^2^)] = 0.036	H-atom parameters constrained
*wR*(*F*^2^) = 0.098	*w* = 1/[σ^2^(*F*_o_^2^) + (0.0466*P*)^2^ + 0.1402*P*] where *P* = (*F*_o_^2^ + 2*F*_c_^2^)/3
*S* = 1.08	(Δ/σ)_max_ < 0.001
1511 reflections	Δρ_max_ = 0.14 e Å^−^^3^
104 parameters	Δρ_min_ = −0.16 e Å^−^^3^
1 restraint	Extinction correction: *SHELXL*, Fc^*^=kFc[1+0.001xFc^2^λ^3^/sin(2θ)]^-1/4^
Primary atom site location: structure-invariant direct methods	Extinction coefficient: 0.105 (9)

### Special details

**Table:**

Geometry. All s.u.'s (except the s.u. in the dihedral angle between two l.s. planes) are estimated using the full covariance matrix. The cell s.u.'s are taken into account individually in the estimation of s.u.'s in distances, angles and torsion angles; correlations between s.u.'s in cell parameters are only used when they are defined by crystal symmetry. An approximate (isotropic) treatment of cell s.u.'s is used for estimating s.u.'s involving l.s. planes.
Refinement. Refinement of *F*^2^ against ALL reflections. The weighted *R*–factor *wR* and goodness of fit *S* are based on *F*^2^, conventional *R*–factors *R* are based on *F*, with *F* set to zero for negative *F*^2^. The threshold expression of *F*^2^ > σ(*F*^2^) is used only for calculating *R*–factors(gt) *etc*. and is not relevant to the choice of reflections for refinement. *R*–factors based on *F*^2^ are statistically about twice as large as those based on *F*, and *R*–factors based on ALL data will be even larger.

### Fractional atomic coordinates and isotropic or equivalent isotropic displacement parameters (Å^2^)

**Table:**

	*x*	*y*	*z*	*U*_iso_*/*U*_eq_	
C1	0.0000	1.0000	0.8502 (3)	0.0603 (7)	
H1	0.0000	1.0000	0.9624	0.072*	
C2	0.0769 (2)	0.91187 (18)	0.7667 (2)	0.0537 (5)	
H2	0.1312	0.8520	0.8215	0.064*	
C3	0.07333 (19)	0.91260 (16)	0.6004 (2)	0.0453 (4)	
C4	0.1444 (2)	0.81022 (17)	0.5072 (2)	0.0564 (5)	
H4A	0.2346	0.7873	0.5610	0.068*	
H4B	0.0806	0.7379	0.5090	0.068*	
C5	0.29756 (19)	0.93047 (18)	0.3315 (3)	0.0590 (5)	
H5A	0.3889	0.8861	0.3158	0.071*	
H5B	0.3040	0.9755	0.4328	0.071*	
C6	0.2762 (2)	1.02106 (18)	0.1970 (2)	0.0567 (5)	
H6A	0.3634	1.0719	0.1855	0.068*	
H6B	0.2618	0.9757	0.0970	0.068*	
C7	0.2098 (2)	0.72780 (17)	0.2461 (3)	0.0617 (5)	
H7A	0.2738	0.6747	0.3093	0.074*	
H7B	0.2628	0.7520	0.1497	0.074*	
C8	0.0801 (3)	0.65392 (18)	0.1972 (3)	0.0671 (5)	
H8A	0.1129	0.5729	0.1595	0.081*	
H8B	0.0193	0.6403	0.2914	0.081*	
N1	0.0000	1.0000	0.5180 (2)	0.0460 (5)	
N2	0.17740 (14)	0.84064 (12)	0.34005 (17)	0.0443 (4)	
O1	0.15472 (15)	1.09903 (12)	0.22517 (18)	0.0608 (4)	
H1A	0.0944	1.0617	0.2802	0.091*	
O2	−0.0051 (2)	0.70862 (16)	0.0769 (2)	0.0867 (5)	
H2A	−0.0531	0.7659	0.1157	0.130*	

### Atomic displacement parameters (Å^2^)

**Table:**

	*U*^11^	*U*^22^	*U*^33^	*U*^12^	*U*^13^	*U*^23^
C1	0.0685 (17)	0.0814 (19)	0.0309 (11)	−0.0226 (16)	0.000	0.000
C2	0.0567 (10)	0.0636 (11)	0.0408 (9)	−0.0147 (9)	−0.0090 (8)	0.0123 (8)
C3	0.0449 (9)	0.0501 (9)	0.0410 (8)	−0.0046 (8)	−0.0020 (7)	0.0090 (7)
C4	0.0620 (11)	0.0540 (10)	0.0533 (11)	0.0119 (9)	−0.0016 (9)	0.0147 (8)
C5	0.0383 (9)	0.0654 (11)	0.0734 (12)	0.0017 (8)	−0.0028 (9)	0.0111 (10)
C6	0.0450 (9)	0.0621 (11)	0.0631 (11)	−0.0064 (8)	0.0069 (9)	0.0084 (10)
C7	0.0612 (11)	0.0542 (10)	0.0696 (12)	0.0205 (10)	0.0148 (10)	0.0044 (9)
C8	0.0867 (14)	0.0460 (10)	0.0687 (12)	0.0053 (10)	0.0155 (10)	−0.0064 (9)
N1	0.0536 (11)	0.0513 (11)	0.0333 (9)	0.0073 (10)	0.000	0.000
N2	0.0406 (7)	0.0433 (7)	0.0491 (8)	0.0064 (6)	0.0038 (6)	0.0053 (6)
O1	0.0550 (8)	0.0577 (7)	0.0697 (9)	0.0026 (6)	0.0056 (6)	0.0142 (7)
O2	0.0996 (12)	0.0797 (11)	0.0807 (10)	0.0185 (9)	−0.0140 (10)	−0.0251 (8)

### Geometric parameters (Å, °)

**Table:**

C1—C2^i^	1.366 (2)	C6—O1	1.410 (2)
C1—C2	1.366 (2)	C6—H6A	0.9700
C1—H1	0.9300	C6—H6B	0.9700
C2—C3	1.380 (3)	C7—N2	1.469 (2)
C2—H2	0.9300	C7—C8	1.482 (3)
C3—N1	1.339 (2)	C7—H7A	0.9700
C3—C4	1.491 (3)	C7—H7B	0.9700
C4—N2	1.455 (2)	C8—O2	1.395 (3)
C4—H4A	0.9700	C8—H8A	0.9700
C4—H4B	0.9700	C8—H8B	0.9700
C5—N2	1.463 (2)	N1—C3^i^	1.339 (2)
C5—C6	1.491 (3)	O1—H1A	0.8200
C5—H5A	0.9700	O2—H2A	0.8200
C5—H5B	0.9700		
			
C2^i^—C1—C2	119.1 (2)	C5—C6—H6A	109.3
C2^i^—C1—H1	120.5	O1—C6—H6B	109.3
C2—C1—H1	120.5	C5—C6—H6B	109.3
C1—C2—C3	119.4 (2)	H6A—C6—H6B	108.0
C1—C2—H2	120.3	N2—C7—C8	115.04 (16)
C3—C2—H2	120.3	N2—C7—H7A	108.5
N1—C3—C2	121.73 (18)	C8—C7—H7A	108.5
N1—C3—C4	117.91 (15)	N2—C7—H7B	108.5
C2—C3—C4	120.25 (18)	C8—C7—H7B	108.5
N2—C4—C3	114.79 (14)	H7A—C7—H7B	107.5
N2—C4—H4A	108.6	O2—C8—C7	114.72 (18)
C3—C4—H4A	108.6	O2—C8—H8A	108.6
N2—C4—H4B	108.6	C7—C8—H8A	108.6
C3—C4—H4B	108.6	O2—C8—H8B	108.6
H4A—C4—H4B	107.5	C7—C8—H8B	108.6
N2—C5—C6	111.48 (16)	H8A—C8—H8B	107.6
N2—C5—H5A	109.3	C3^i^—N1—C3	118.6 (2)
C6—C5—H5A	109.3	C4—N2—C5	110.45 (16)
N2—C5—H5B	109.3	C4—N2—C7	111.27 (14)
C6—C5—H5B	109.3	C5—N2—C7	111.38 (15)
H5A—C5—H5B	108.0	C6—O1—H1A	109.5
O1—C6—C5	111.42 (17)	C8—O2—H2A	109.5
O1—C6—H6A	109.3		
			
C2^i^—C1—C2—C3	−1.04 (13)	C4—C3—N1—C3^i^	175.19 (18)
C1—C2—C3—N1	2.2 (3)	C3—C4—N2—C5	70.9 (2)
C1—C2—C3—C4	−174.02 (14)	C3—C4—N2—C7	−164.87 (16)
N1—C3—C4—N2	23.7 (2)	C6—C5—N2—C4	−143.71 (17)
C2—C3—C4—N2	−160.01 (17)	C6—C5—N2—C7	92.11 (19)
N2—C5—C6—O1	66.6 (2)	C8—C7—N2—C4	77.8 (2)
N2—C7—C8—O2	71.9 (2)	C8—C7—N2—C5	−158.52 (17)
C2—C3—N1—C3^i^	−1.09 (13)		

Symmetry codes: (i) −*x*, −*y*+2, *z*.

### Hydrogen-bond geometry (Å, °)

**Table:**

*D*—H···*A*	*D*—H	H···*A*	*D*···*A*	*D*—H···*A*
O2—H2A···O1^i^	0.82	1.94	2.762 (2)	175
O1—H1A···N2^i^	0.82	2.74	3.248 (2)	122
O1—H1A···N2	0.82	2.54	2.935 (2)	111
O1—H1A···N1	0.82	2.25	3.004 (2)	153

Symmetry codes: (i) −*x*, −*y*+2, *z*.
